# Dilemmas and deliberations in managing the care trajectory of elderly patients with complex health needs: a single-case study

**DOI:** 10.1186/s12913-022-08422-3

**Published:** 2022-08-12

**Authors:** Marianne Kumlin, Geir Vegar Berg, Kari Kvigne, Ragnhild Hellesø

**Affiliations:** 1 Department of Health and Nursing Sciences, Inland Norway University of Applied Sciences, Elverum, Norway; 2 Innlandet Hospital Trust, Lillehammer, Norway; 3 Department of Nursing Science, Institute of Health and Society, Faculty of Medicine, University of Oslo, Oslo, Norway; 4 Department of Health Sciences, Faculty of Medicine and Health Sciences, Norwegian University of Science and Technology Gjøvik, Gjøvik, Norway

**Keywords:** Elderly, Care trajectory, Complex healthcare needs, Person centred, Coherence

## Abstract

**Background:**

Today, the ageing population is larger than ever before, and people who are living longer with chronic illnesses and multimorbidity need support from multiple healthcare service levels. Similarly, healthcare systems are becoming increasingly specialised and fragmented. The World Health Organization has highlighted novel policies for developing integrated and person-centred services. However, patients, next of kin and health professionals face several challenges in managing healthcare during the care trajectory. Limited literature has addressed the challenges experienced by these groups. Therefore, this study aimed to identify the dilemmas and deliberations faced by patients, next of kin and health professionals during the care trajectory of elderly patients with complex healthcare needs.

**Method:**

The study had a qualitative single-case design. The case was taken from a multi-case study exploring the care trajectory of elderly patients. The participants were the patient, their next of kin and the health professionals involved in the patient’s care trajectory. Data were obtained via observation and individual interviews conducted during the patient’s hospital stay and after the patient returned home.

**Results:**

The dilemmas and deliberations in managing the care trajectory were divided into four main themes: the health professionals’ pursuit of appropriate and feasible healthcare services, the next of kin’s planning horizons, being the person left in limbo and reorganising the home for comprehensive healthcare.

**Conclusion:**

The pursuit of a tailored and suitable healthcare service lead to a comprehensive mobilisation of and work by all actors involved. Having a comprehensive understanding of these conditions are of importance in developing an appropriate care trajectory for the elderly patient with complex need.

## Background

Globally, the mean age of the population is increasing, and within this ageing population, more people are living longer with chronic health conditions and multimorbidity [1, 2]. When these people encounter healthcare systems that are becoming more specialised and fragmented, they must receive healthcare from multiple professionals who work at different levels of the healthcare services [3, 4]. To establish coherence for elderly people with complex healthcare needs, care pathways have been developed with the aims of delivering professional and efficient care, controlling resources and improving patient satisfaction and the predictability of the patient’s trajectory [5–7]. A key feature is to achieve a streamlined process in a patient’s trajectory and to prevent undesirable events.

Research has shown that while care pathways are most effective when patients’ care trajectories are foreseeable and when directed towards single diseases [7, 8], care pathways developed for single diseases have limited ability to meet the individual and complex needs of patients with multimorbidity [4, 5, 9–11]. These patients are often in need of care from several health professionals, both from primary and specialist healthcare services [4]. There is a risk of breaches of coherence and continuity of care when patients require services from multiple levels with many actors; hence, patients with multimorbidity are more vulnerable to healthcare service fragmentation, and they experience less continuity of care than patients with a single condition [4, 12].

Inadequate care coordination negatively affects the care experience and patient outcome [13, 14], and it has been shown that elderly people and their next of kin experience insufficient coherence in healthcare services and fragmentation in care delivery [15, 16].

A person-centred approach aligns the healthcare services with the values, needs and desires of the patient and reinforces the importance of involving patients in discussions and decisions [17]. However, patients experience to be inadequately involved in the decision-making about their own healthcare needs [18–20]. It has also been found that patients and their next of kin experience a high workload when they attempt to achieve coherence in their care trajectory [21–23]. Furthermore, patients with chronic conditions and their next of kin are expected to constantly attend to managing and changing their daily routines as part of treatment or healthcare. This work not only includes tasks at home, but also comprehensive planning and coordinating of healthcare [16, 24, 25].

An increasing awareness of the limitations of care pathways for patients with multimorbidity has led to efforts to develop more generic patient care pathways and strategies to enhance care coordination; the focus is on being more flexible and adaptable to the needs of individuals with multimorbidity [9, 26–29]. Despite the many strategies currently available for developing interdisciplinary care pathways, achieving inter-professional collaborations that deliver coherent and seamless healthcare is still far from complete [30].

In Norway, where the current study was conducted, the healthcare system is a two-level model. The national authorities organise and manage specialist care, governing via policy and professional guidelines [31]. The municipalities govern the primary care services, which encompass home healthcare services, nursing homes, rehabilitation, public health and general practitioners (GPs). These are governed by local authorities. In 2012, to improve the deficiencies identified (namely discontinuity and disintegrated care) across these levels of healthcare, the Norwegian government launched a coordination reform to develop coordinated care pathway across primary and specialist healthcare. With this reform, the municipalities gained more responsibility for overall healthcare and rehabilitation needs, with the aim of decreasing the length of hospital stay. The reform also regulated the municipalities’ payment arrangement: municipalities began being charged when there were ready-to-discharge patients remaining in the hospital who were waiting for a municipal offer.

Patients’ considerations of their own healthcare needs can be an ongoing issue during their entire care trajectories [32]. Moreover, health professionals have specified the challenges associated with offering coherent healthcare for elderly patients [33]. Much research has been conducted to investigate the interactions between and integration of specialised and fragmented healthcare services [16]. Even though there is ongoing research on developing more generic and integrated care pathways for patients needing complex care [10, 16, 34–36], few studies have addressed the involved actors’ dilemmas and deliberations during the care trajectory of elderly patients with complex and comprehensive healthcare needs.

### Aim

The aim of this study was to illuminate the dilemmas and deliberations of patients, next of kin and health professionals during the care trajectory of elderly patients with complex healthcare needs.

## Design and methods

To gaining an in-depth understanding of the underlying mechanisms behind the problems that occur during a patient’s care trajectory, we applied a single-case design. The case was taken from a multi-case study exploring the care trajectories of elderly patients and how they participate in and interact within their care trajectory across various healthcare systems [32]. The case was selected because it contains comprehensive data relevant to the topic of the study, namely the complexity of a care trajectory and dilemmas encountered and deliberations made by the involved actors. The individual stories formed a crucial aspect of the findings and provided an opportunity to achieve more complete knowledge and a comprehensive understanding of the case [37].

Utilising multiple data sources is advisable in case studies because it allows diverse perspectives to be captured [38]. The present case included data from participant observation, individual interviews and the patient record system.

### Setting and participants

This study was initiated at a hospital in an urban area. Further along the trajectory, the study took place in the patient’s home municipality, which has a larger population size. The healthcare services in this municipality were specialised and differentiated, as is the tendency today in municipalities with larger population sizes. In comparison, smaller municipalities often have a more generalist approach [39]. The physical distance between the patient’s home and the hospital was less than 20 km.

### Participants

The described participants are the actors involved in the selected case (Table 1). The recruitment process started with a contact nurse in the hospital department who delivered verbal and written information about the study to the patient. When the patient consented to participate, the information was repeatedly delivered to the patient by the first author, and the patient signed a consent form. Furthermore, the patient was asked for consent to contact his next of kin and the healthcare personnel involved in his care trajectory. The patient was also asked for permission to extract his data from the patient record system. After receiving these permissions, the first author contacted and provided verbal and written information to the next of kin and health professionals included in the study.

**Table 1:** Participants included in this case

Participant		Number of participants
Patient	Albert (aged above 80)	1
Next of kin	Spouse	1
	Son	1
Hospital health professional	Nurse	4
	Physiotherapist	1
	Doctor	1
Municipal health professional	Nurse	3
	Physiotherapist	1
	Occupational therapist	1

### Data collection

The data collection was conducted during the spring of 2018. The first author carried out participant observations for 2 weeks. The observations were initiated on the day the patient was discharged from the hospital and included the patient’s journey back home, as well as the reception and meetings with the interdisciplinary team and home care nurses in the patient’s home. Additional observations occurred at the patient’s home and the health professionals’ offices during workdays. During the observation period, interviews with the patient, next of kin, and health professionals were performed. To extend the understanding of what had occurred during the care trajectory, the main themes and topics of the interview covered events before hospitalisation, the current situation and future perspectives on the care trajectory. Data from the interviews and documentation from the patient’s records were gathered to investigate and obtain insight into the period from admission to discharge from the hospital. Furthermore, the first author participated in a meeting in which professionals from the hospital and the municipality discussed their collaboration. Field notes were taken to document the observations. During the observations, only short sentences and key words were written down. More extensive notes were recorded immediately after an observation. Short dialogues with the participants were recorded as field notes.

### Analysis

Field notes, transcribed interviews and documents extracted from the patient record were organised chronologically through the trajectory to coherently comprehend the case’s story [38]. All the data were assessed as a complete set and read as a whole several times in search of patterns and themes [40]. Initially, descriptive and analytical codes were generated. Subsequently, subthemes and preliminary themes representing the data were generated. Development of the main themes was guided by the research question. The entire analytical process alternated between the various data sources to investigate, justify, supplement or expand the insight and understanding of the data material along with the study’s objective [41].

### Ethical considerations

The Norwegian Centre for Research Data was notified about the study (ID: 54551) and determined that the study did not raise any ethical issues that needed ethical approval. The Institutional Review Board at the hospital also assessed and approved the study. Participation in the study was voluntary and based on informed consent given both verbally and in writing. During the observation period, special attention was paid to ensure that the patient was participating voluntarily and with informed consent, and information about the study and voluntary participation was repeatedly provided. Information about the possibility of withdrawing any time from the study was given both verbally and in writing. To ensure protection of the participants’ anonymity, some of the demographic data were rewritten.

### Findings

The main themes derived from the analysis were illuminated through the account of the care trajectory case. The themes were as follows; the healthcare professionals’ pursuit for appropriate and feasible health care services, the next of kin’s planning horizons, being the person left in limbo and reorganizing the home for comprehensive healthcare. Initially we will present the case Albert.

Albert resided in a house in the countryside with his spouse. He suffered from several chronic diseases and received daily home care for a chronic ulcer and for help with medication administration. One day, he fell in the garden and was admitted to the hospital, where he was diagnosed with a hip fracture. Albert was immediately entered into a care pathway devised for patients with hip fractures, named the fast-track programme. This standardised and quality assured programme provided him with professional assessments during the preoperative phase and decreased the time he had to wait for surgery. The objective of the programme is to initiate the required surgery within 24 hours to prevent unnecessary complications. It includes patient examination in the ambulance, fast diagnosis upon arrival at the emergency department and immediate preoperative care [42, 43]. Albert arrived at the emergency department in the evening, was immediately diagnosed and given preoperative care, and had prosthetic surgery the following morning. Hence, this part of his care trajectory was successful according to the objectives of the fast-track programme [43].

Elective prosthetic surgery patients at the hospital Albert was admitted typically enter a standardised care pathway before surgery. They are invited to participate in a ‘prosthetic school’ before being admitted to the hospital. The standardised care pathway accelerates the patients’ recovery by including immediate training after the surgery and after discharge from the hospital. The patients should receive physiotherapy in their home municipality after discharge from the hospital, followed by a control at the hospital. This care pathway for prosthetic surgery patients is based on a short-term and effective care trajectory.

Emergency patients with a hip fracture, such as Albert, are offered a physiotherapy programme in the hospital through the fast-track programme. No other standardised care pathway applies to these patients. However, the hospital does have ‘rehabilitation beds’ for patients who are self-reliant in daily activities but need additional rehabilitation support. Albert required further rehabilitation after his surgery; however, he was ineligible for a rehabilitation bed because his healthcare needs were too complicated. A hospital nurse stated:

‘Some patients “fall between two chairs”. Some complications occur; those who have fallen and are frail need a rehabilitation bed.’

A physiotherapist noted the differences in the follow-up for the prosthetics patients as follows:

‘The length of hospital stay has decreased dramatically. The hospital does not want us to use the rehabilitation bed because they have to pay for it. For the oldest patients with a hip fracture and prosthetic surgery, there is no control at the hospital. The municipality should do the follow-up. We do not know exactly how it goes with the oldest.’

When Albert was deemed ineligible for a hospital rehabilitation bed due to his comprehensive care needs post-surgery, the complex and unforeseen part of his care trajectory began.

### The health professionals’ pursuit of appropriate and feasible healthcare services

When it became obvious that Albert was too sick and vulnerable to continue in the planned care pathway, several dilemmas arose that were associated with finding the best options for meeting his ongoing healthcare needs. While the municipal health professional was assessing suitable rehabilitation options, the hospital health professionals determined that, due to hospital policy, Albert had to be moved to an outside bed in another unit at the hospital during the waiting time. The outside bed was used for patients who could not be discharged according to plan, and for patients assessed as ready for discharge who were waiting for an offer from the municipality. The health professionals had the responsibility of deciding which patients were eligible for relocation to an outside bed.

Albert stayed at the initial unit for 5 days before being transferred to an outside bed. The professionals articulated that they strived to ensure the quality of care by stating that all patients would stay in the initial unit for at least the first 1 or 2 days. The physicians from the initial unit continued to follow-up patients moved to an outside bed. The nurses expressed that relocating the patient usually resulted in inferior follow-up and meant less professional care and less continuity of care. A nurse described how challenging it was to keep patients in the initial unit and to make assessments regarding the most suitable patients to move:

‘All units must provide an overview of their bed capacity. We try to put the right patient in the right place. For some patients there has been a lot of changes. We make a list of the most eligible patients to be moved from the initial unit.’

The day after Albert was moved to the outside bed, he was considered ready for discharge. The municipality offered him a rehabilitation bed at the municipality health centre, and he was transferred the same day. However, on the first evening at the municipality health centre, Albert fell again. This is what his son said about Albert’s first hour at the centre:

‘At the health centre, he only stayed for seven or eight hours. My mother and I travelled back home at five pm, and at nine pm in the evening they called me and told me he had fallen again. My father expressed that he wanted to go home.’

Albert’s health condition was assessed as being too complex for further care at the health centre, and he was re-admitted to the same hospital.

The hospital’s bed policy influenced the municipal health professionals’ deliberations while they were searching for the best healthcare service for Albert. The hospital’s policy of emptying beds was highly challenging for the municipal health professionals because they already had issues with the capacity in the municipality. It was considered challenging to ensure sufficient turnover in the municipality’s short-term beds, rehabilitation beds and long-term beds. The health professionals expressed that these beds were too often ‘blocked’ by patients in need of long-term care, making it a continuous challenge to find an empty bed when needed. Here is a description provided by one of the nurses:

‘Because those [patients] who get a decision on long-term care are blocking short-term beds, so right now … from last week until today, there are some short-term beds available, but now patients who receive homecare and need to get a “boost” for nutritional follow-up and caring are placed in these beds. It’s rolling, but it is just a matter of time when it blocks again.’

The challenges associated with effective bed use were evident during a dialogue between the municipal and hospital health professionals on Albert’s planned discharge date. On day eight, Albert was considered ready for discharge from the hospital, and the municipality arranged a rehabilitation bed for him. However, the hospital physicians decided to perform an additional medical evaluation before discharging. The intense exchange of information on the discharge day between the hospital and municipality is reflected in the digital messaging samples shown in Table 2.

**Table 2:** Example of electronic message communication between the hospital and municipal health professionals on Albert’s discharge day

Message sender	Time	Message
**Municipality**	10.45	Thanks for the information. Possibly he will get a place at a health centre tomorrow – for a short-term rehabilitation stay. You will finally be informed this morning.
**Hospital**	10.50	Notification of patient ready for discharge has been sent. I hope he gets a bed at the health centre.
**Hospital**	10.56	De-registration of a patient ready for discharge is sent. The patient waits for a medical assessment before he is ready to be discharged.
**Municipality**	11.02	He gets a place/bed at the health centre tomorrow.
**Hospital**	13.40	Notification of patient ready for discharge sent. The patient has received medical supervision and is assessed ready for discharge.
**Municipality**	14.36	Because he was de-registered ready to be discharged earlier today, another patient has been allocated. Therefore, we do not have room for him until next week.

When the municipality received the de-registration, the health professional working there decided to offer the rehabilitation bed initially intended for Albert to another waiting patient. This launched a new search for a rehabilitation bed, which ended in Albert eventually being offered a rehabilitation bed in another municipality.

**Fig. 1:**
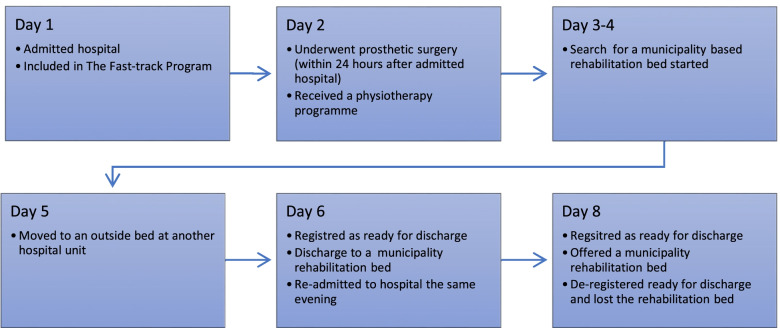
Timeline of events from admission to the hospital and covering the subsequent 8 days

**Fig. 2:**
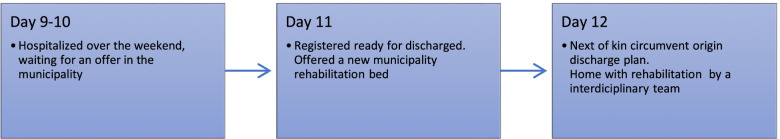
Timeline of events from day nine at the hospital until day twelve

Figures 1 and 2 illustrate Albert’s care trajectory. The entire timeline is shown, from his initial admission to the hospital to the time he was discharged and returned to his home.

### The next of kin’s planning horizons

During his entire hospital stay, Albert’s next of kin were in the background, and they played an active role in the planning, which eventually affected the decision about Albert’s discharge location. In line with the health professionals’ pursuit of a rehabilitation bed during the care trajectory, the next of kin had their own planning horizons. They constantly followed the planning process and weighed up the different decisions against what they believed would be best for the patient. With regard to the conversations and negotiations among the hospital and municipal health professionals, the next of kin noted that they had been regularly informed. They were concerned about whether Albert would be sent home and worried about Albert being discharged too soon. They believed that Albert was not ready and strove to postpone the discharge. Albert’s son described how they sought to balance what they thought was best for the patient against what the healthcare services had to offer:

‘I had some phone calls with the staff at the hospital. He [my father] had been ready for discharge for a while … and they wanted to send him home several times, but we told them that we cannot take him home yet, as my mother is 87 years old, … but I understand that if a patient is ready for discharge, it costs a lot to stay in the hospital and they want the patient out, but …’

After re-admission to the hospital, the next of kin searched for an alternative rehabilitation plan. Albert was offered a rehabilitation bed in a nursing home in the neighbouring city within a few days. The next of kin considered this nursing home to be too far away from his home and shared that it would become difficult to visit Albert, especially for his wife. Thus, they considered possibilities for rehabilitation at home. The day before Albert was discharged from the hospital to a nursing home, the original plan was dismissed, and it was decided that he would return to his home. The next of kin drew up a plan with the municipal health professionals for reorganising the house and securing the necessary aids. This alternative plan involved facilitating Albert’s rehabilitation and follow-up care via an interdisciplinary team and home care nurses.

### Being the person left in limbo

While the hospital and municipal personnel were devising a possible and feasible rehabilitation alternative, the patient seemed to be left in limbo during this part of the planning. He became the person who was at the centre of all the deliberations, yet not involved in the decisions. This became more evident on the day of discharge.

On the morning of the discharge day, it was unclear whether Albert was ready for discharge or not. Moreover, he was unsure about whether he was going home or to a nursing home. He described his assumptions about what should have happened next as follows:

‘Yes, today, my children will probably meet at home and then move the inventory from the first floor down to the ground floor. There, we have a bathroom and all, which is simple. The youngest should pick me up today and drive me home.’

The issues related to discharge were ongoing close to Albert’s expected departure time. In line with this process, various activities needed attention, as exemplified in the following field note:

‘In the morning, a nurse entered the room to weigh Albert. She told him that she should weigh him because he might be going home today—no other explanations. He did not respond to the nurse but placed himself on the chair scales and waited for what would be next. Then, with Albert still sitting on the chair scales, a new nurse entered the room and verified that he could go home today.’

When the ready-for-discharge decision was finally made, Albert did not have adequate time to make himself ready to leave the hospital. Yet one hour later, he was in a taxi and on his way home. This was recorded in another field note:

‘Everything seemed to be done quickly; among other things, Albert’s personal belongings had to be packed. The nurse had just finished with his documents when the taxi driver arrived. Albert did not say anything but kept attention to what was happening. On the way out of the door, he asked for his box of chocolates and the newspaper. He ensured that he had them.’

When Albert arrived at his home, his next of kin and two people from a municipal interdisciplinary team were there**.** The house had been fitted with relevant appliances, and Albert’s bed had been moved down into the living room. Albert’s first hours at home included several activities, and healthcare was the main issue. One of the health professionals called it the ‘reception day’. Albert had to respond to questions and instructions from several health professionals from various work teams. The following field note describes some of these activities and what occurred during this afternoon on the discharge day:

‘Albert managed out of the car and got into the wheelchair with the driver’s help. The son and two from the interdisciplinary team were outside the house. His spouse was in the hallway welcoming him. He had a new wheelchair to use inside the house. Before he wheeled into the living room, Albert stopped and made sure that he had paid for the taxi. Someone from the interdisciplinary team enquired if Albert could put weight on the leg in the living room and checked the record. The team soon started testing how he could stand up and walk using a walking frame and show different techniques. The son was worried about all the things his father needed to remember. Albert seemed to manage the instruction. Albert asks for how long he has been away and once tries to get up from the wheelchair with the help of a walker. It goes well, and he looks satisfied. Further, the team informs about the social alarm and how it works. The social alarm has yet to be delivered.’

Before the day ended, Albert met with two more professional teams. A nurse from the nursing team came to remove an intravenous catheter and evaluate the further need for nursing. Later in the evening, a nurse from the home care service came to help Albert with his mobilisation and to get dressed for the night.

### Reorganising the home for comprehensive healthcare

The decision to set up a rehabilitation bed in the patient’s home resulted in extensive work for Albert, his next of kin and several health professionals from the interdisciplinary team, the nursing team and the home care services.

Before Albert arrived home, his next of kin had rearranged his bedroom and moved his bed from the first floor to the living room on the ground floor, where various aids could be installed. They expressed that, by reorganising the furniture, they had modified the home to allow for the provision of healthcare services. The reason for reorganising was that the home should be a residence and a suitable place for Albert during his rehabilitation phase, as well as an appropriate working place for the professional teams. However, Albert’s spouse shared that she was not so eager about modifying the home. For her, it was a dilemma that she had to sleep in the living room after the beds were moved down from the bedroom. The following is a statement by Albert’s spouse:

‘I don’t like modifying our home; I want it to look nice. But, I know it is just temporary.’

Organising Albert’s healthcare included planning various activities, preparing different schedules and assuring work quality. Action lists, report systems and deviations needed coordination. A health professional in the interdisciplinary team said:

‘We come when the homecare service has finished their work. When the patient has received food and is dressed; we move into exercise, all the daily activities and muscles’ strengthening and movement.’

The extensive scheduling coordinated the activities of not only the various professional teams but also Albert and his next of kin. His next of kin reported how they divided tasks among themselves and decided who was best to do what. They described a 24/7 schedule, where someone was always on duty, both at the hospital and when Albert arrived home. Albert’s next of kin described this experience as follows:

‘All the time, there is something on “my shoulder”, anywhere you go, you worry and think about being in another place. If we travel away, the other family members are responsible for looking after them and taking a visit after their job or a call. We do not travel away all at the same time. That will not work—oh no, that will not work.’

It became obvious that the professionals’ schedule was challenging for Albert. He was forced to adapt to various professionals’ schedules and working methods. However, in his own way, Albert was able to take some control of the situation. For example, when being helped with getting dressed, he slowed down and performed the activities at his own pace. A nurse from the home care service shared that they are allocated specific time for each patient and noted that the number of patient visits during a work shift could be as high as 30. Still, it was impossible to increase the tempo during her time with Albert. Following Albert’s rhythm was essential and this took time, despite the nurse’s schedule. The nurse commented on this situation as follows:

‘Things have clearly changed for Albert; now he needs help with getting dressed. It will now take more time. I cannot follow the listed work time because it is unrealistic.’

## Discussion

The main findings in this study show how the pursuit of a suitable healthcare service within a tailored care trajectory resulted in several dilemmas, considerable deliberations and a comprehensive mobilisation of work for all the involved actors. The search for a suitable healthcare service lasted throughout the entire trajectory, from when the patient went to the hospital until he returned home.

The expectations of efficient healthcare services and the emptying bed policy had a domino effect throughout the patient’s care trajectory. Therefore, a dilemma arose for health professionals, who had to negotiate about the use of resources and the coordination of healthcare services, instead of focusing on delivering a person-centred approach [17, 44].

When Albert had occupied a hospital bed, he practically become a ‘bed-blocker’. The term ‘bed blocking’ has been used to describe the situation when a patient is ready for discharge from the hospital but is waiting for admission to a municipal nursing home or for municipal-managed home care [45]. We found that bed blocking is not specific to hospitals but also occurs in the municipality. A municipal health professional described how they strove to ensure that there was a sufficient turn-over of short-term, rehabilitation and long-term beds.

A tension about who held the actual decision-making power became visible during the discourse between the health professionals at the hospital and municipality. The parallel searches for an available bed resulted in several negotiations between the different parties, and both sides had different ‘cards’ to play. While the hospital health professionals had the authority to decide about the patient’s readiness for discharge and register them as ready for discharge, the municipal health professionals had access to bed capacity in the municipality. The need for quick solutions and decisions about the bed capacity in the discharge planning sped up the decision-making process and influenced the communication among the health professionals. It is problematic that structural factors, such as available time, can impact health professionals’ work and their interactions to achieve coherence with the patient’s needs [30, 46–48].

The patient was left in limbo during the back-and-forth process in the healthcare system. A structured care trajectory designed to ensure that patients receive optimal treatment and avoid complications was followed by an unpredictable course for the patient. Given the complexity of managing the healthcare trajectory, the patient had almost no say in the decisions, causing him to be left in limbo. Therefore, it is appropriate to pose the following legitimate questions: Was the situation too complex for the health professional to include the patient in the plans? Was there a time and place for including the patient in the plans [30, 48]?

Today, it is expected that patients will participate in discussions and decisions about their healthcare, taking an active role, and that healthcare services be considered players in the system [49]. We found that this may create a dilemma for patients; the ideal of participation is weighed down by the attendant facilitation. For Albert, it seems that his involvement in the decisions about his trajectory was limited. The challenges to providing health care services supposed to involve the elderly persons in decisions about their healthcare is in line with previous research [50]. Albert chose to trust his next of kin to support him in the planning and the making of decisions. Research has shown that patients delay their decisions when they feel they are not ready to make them during the care trajectory [32].

The present study’s findings also indicated that preparing for and undergoing rehabilitation at home can require a comprehensive mobilisation to reorganise the patient’s home. Several health professional teams participated in this mobilisation of rehabilitation care. In this comprehensive mobilisation of care trajectory, the next of kin worked in the background and contemplated the alternatives alongside the health professionals. Next of kin wanted to provide assurance and confidence during the discharge process and after the discharge from the hospital and other transitions in the healthcare system. They also considered what could be best for the patient in their life [22, 51].

The next of kin undertake their various critical tasks under conditions that are different from those experienced by the health professionals. In this case, they had limited influence on the healthcare services decisions and lacked access to information and resources. Thus, they had to adapt to the healthcare system rather than the other way around. They strove to organise their lives and jobs around caring for their relative. Studies on family care for the elderly have stressed the need for flexible hours, schedules and locations to allow carers to manage both jobs and elder care [21]. In the ageing population, family care contributes to filling healthcare gaps [23], and efforts are underway to optimise familial care capacity in many countries. Clearly, there is a need to identify and understand the work and role of the next of kin to examine the actual coherence with policy and expectations.

## Methodological strengths and limitations

With her pre-understanding of the topic, the first author (MK), an experienced geriatric nurse, might have affected the observations and interviews. However, her experience and knowledge contributed to the understanding of the research field and to the input during data collection. The study has a limitation in that the observations began on the discharge day at the hospital. Data for the period between hospital admission and discharge were therefore sourced from interviews and documentation from the patient’s record. The use of a single case provided a deeper insight into dilemmas and deliberations during the care trajectory of elderly patients and what occurred when the patient’s care trajectory deviated from the standardised condition. Thus, this approach increased the value of the present study. A single case can shed light on several larger classes of cases [52].

## Conclusion

In this study, where the aim was to illuminate the dilemmas and deliberations of patients, next of kin and health professionals in managing the care trajectory, we found that health professionals strove between negotiation about allocation of resources and being person-centred. The pursuit for tailored healthcare services leads to a comprehensive mobilisation of and work by all the actors across the entire care trajectory. The next of kin sought to balance the patient’s needs with the offered healthcare services and the patient’s participation in the planning and decisions was limited. Identifying the conditions and work involved is important in developing appropriate care trajectories that align in coherence with complex healthcare needs of elderly patients.

## Data Availability

The datasets generated during the current study are not publicly available due to the sensitive and identifiable nature of the data but are available from the corresponding author on reasonable request.

## Abbreviation

GP: General practitioner

## References

[1] James SL, Abate D, Abate KH, Abay SM, Abbafati C, Abbasi N (2018). Global, regional, and national incidence, prevalence, and years lived with disability for 354 diseases and injuries for 195 countries and territories, 1990–2017: a systematic analysis for the global burden of disease study 2017. Lancet.

[2] Storeng SH, Øverland S, Skirbekk V, Hopstock LA, Sund ER, Krokstad S (2022). Trends in disability-free life expectancy (DFLE) from 1995 to 2017 in the older Norwegian population by sex and education: the HUNT study. Scand J Public Health..

[3] Chatterji S, Byles J, Cutler D, Seeman T, Verdes E (2015). Health, functioning, and disability in older adults—present status and future implications. Lancet.

[4] Doessing A, Burau V (2015). Care coordination of multimorbidity: a scoping study. J Comorbid.

[5] De Bleser L, Depreitere R, De Waele K, Vanhaecht K, Vlayen J, Sermeus W (2006). Defining pathways. J Nurs Manag.

[6] McLachlan S, Kyrimi E, Dube K, Hitman G, Simmonds J, Fenton N (2020). Towards standardisation of evidence-based clinical care process specifications. Health Inform J.

[7] Aspland E, Gartner D, Harper P (2021). Clinical pathway modelling: a literature review. Health Systems.

[8] Vanhaecht K (2007). The impact of clinical pathways on the organisation of care processes.

[9] Røsstad T, Garåsen H, Steinsbekk A, Sletvold O, Grimsmo A (2013). Development of a patient-centred care pathway across healthcare providers: a qualitative study. BMC Health Serv Res.

[10] Berntsen G, Strisland F, Malm-Nicolaisen K, Smaradottir B, Fensli R, Røhne M (2019). The evidence base for an ideal care pathway for frail multimorbid elderly: combined scoping and systematic intervention review. J Med Internet Res.

[11] Moser A, Melchior I, Veenstra M, Stoffers E, Derks E, Jie KS (2021). Improving the experience of older people with colorectal and breast cancer in patient-centred cancer care pathways using experience-based co-design. Health Expect.

[12] Pearson-Stuttard J, Ezzati M, Gregg EW (2019). Multimorbidity—a defining challenge for health systems. Lancet Public Health.

[13] Haland E, Rosstad T, Osmundsen TC (2015). Care pathways as boundary objects between primary and secondary care: experiences from Norwegian home care services. Health (London).

[14] Amelung V, Stein V, Goodwin N, Balicer R, Nolte E, Suter E (2017). Handbook integrated care.

[15] Gallagher N, MacFarlane A, Murphy AW, Freeman GK, Glynn LG, Bradley CP (2013). Service users’ and caregivers’ perspectives on continuity of care in out-of-hours primary care. Qual Health Res.

[16] Lawless MT, Marshall A, Mittinty MM, Harvey G (2020). What does integrated care mean from an older person’s perspective? A scoping review. BMJ Open.

[17] Constand MK, MacDermid JC, Dal Bello-Haas V, Law M (2014). Scoping review of patient-centered care approaches in healthcare. BMC Health Serv Res.

[18] Efraimsson E, Rasmussen BH, Gilje F, Sandman P (2003). Expressions of power and powerlessness in discharge planning: a case study of an older woman on her way home. J Clin Nurs.

[19] Nyborg I, Danbolt LJ, Kirkevold M (2017). User participation is a family matter: a multiple case study of the experiences of older, hospitalised people and their relatives. J Clin Nurs.

[20] Sivertsen DM, Lawson-Smith L, Lindhardt T (2018). What relatives of older medical patients want us to know-a mixed-methods study. BMC Nurs.

[21] Bookman A, Harrington M (2007). Family caregivers: a shadow workforce in the geriatric health care system?. J Health Polit Policy Law.

[22] Wittenberg Y, Kwekkeboom R, Staaks J, Verhoeff A, de Boer A (2018). Informal caregivers’ views on the division of responsibilities between themselves and professionals: a scoping review. Health Soc Care Commun.

[23] Fast J, Keating N, Eales J, Kim C, Lee Y (2021). Trajectories of family care over the lifecourse: evidence from Canada. Ageing Soc.

[24] Mattingly C, Grøn L, Meinert L (2011). Chronic homework in emerging borderlands of healthcare. Cult Med Psychiatry.

[25] Kvæl LAH, Debesay J, Bye A, Bergland A (2020). The dramaturgical act of positioning within family meetings: negotiation of patients’ participation in intermediate care services. Qual Health Res.

[26] Grimsmo A (2018). Antall kroniske sykdommer og persontilpasning bør ligge til grunn for prioriteringer i kommunale helse-og omsorgstjenester. Tidsskrift for omsorgsforskning.

[27] Skrove GK, Bachmann K, Aarseth T (2016). Integrated care pathways—a strategy towards better care coordination in municipalities? A qualitative study. Int J Care Coord.

[28] Everink IH, van Haastregt JC, Tan FE, Schols JM, Kempen GI (2018). The effectiveness of an integrated care pathway in geriatric rehabilitation among older patients with complex health problems and their informal caregivers: a prospective cohort study. BMC Geriatr.

[29] Saltvedt I, Prestmo A, Einarsen E, Johnsen LG, Helbostad JL, Sletvold O (2012). Development and delivery of patient treatment in the Trondheim hip fracture trial. A new geriatric in-hospital pathway for elderly patients with hip fracture. BMC Res Notes.

[30] Schot E, Tummers L, Noordegraaf M (2020). Working on working together. A systematic review on how healthcare professionals contribute to interprofessional collaboration. J Interprof Care.

[31] Sogstad M, Hellesø R, Skinner MS (2020). The development of a new care service landscape in Norway. Health Services Insights.

[32] Kumlin M, Berg GV, Kvigne K, Hellesø R (2020). Elderly patients with complex health problems in the care trajectory: a qualitative case study. BMC Health Serv Res.

[33] Kumlin M, Berg GV, Kvigne K, Hellesø R (2021). Unpacking healthcare professionals’ work to achieve coherence in the healthcare journey of elderly patients: an interview study. J Multidiscip Healthc.

[34] Rosstad T, Garasen H, Steinsbekk A, Haland E, Kristoffersen L, Grimsmo A (2015). Implementing a care pathway for elderly patients, a comparative qualitative process evaluation in primary care. BMC Health Serv Res.

[35] Røsstad T, Salvesen Ø, Steinsbekk A, Grimsmo A, Sletvold O, Garåsen H (2017). Generic care pathway for elderly patients in need of home care services after discharge from hospital: a cluster randomised controlled trial. BMC Health Serv Res.

[36] Grimsmo A, Lohre A, Rosstad T, Gjerde I, Heiberg I, Steinsbekk A (2018). Disease-specific clinical pathways - are they feasible in primary care? A mixed-methods study. Scand J Prim Health Care.

[37] Flyvbjerg B (2006). Five misunderstandings about case-study research. Qual Inq.

[38] Patton MQ (2014). Qualitative Research & Evaluation Methods 2021.

[39] Rostad HM, Skinner MS, Hellesø R, Sogstad MKR (2020). Towards specialised and differentiated long-term care services: a cross-sectional study. BMC Health Serv Res.

[40] Braun V, Clarke V (2006). Using thematic analysis in psychology. Qual Res Psychol.

[41] Morgan SJ, Pullon SRH, Macdonald LM, McKinlay EM, Gray BV (2017). Case study observational research: a framework for conducting case study research where observation data are the focus. Qual Health Res.

[42] Gomez M, Marc C, Talha A, Ruiz N, Noublanche S, Gillibert A (2019). Fast track care for pertrochanteric hip fractures: how does it impact length of stay and complications?. Orthopaed Traumatol Surg Res.

[43] Wallace R, Angus L, Munnangi S, Shukry S, DiGiacomo JC, Ruotolo C (2019). Improved outcomes following implementation of a multidisciplinary care pathway for elderly hip fractures. Aging Clin Exp Res.

[44] McCormack B, Karlsson B, Dewing J, Lerdal A (2010). Exploring person-centredness: a qualitative meta-synthesis of four studies. Scand J Caring Sci.

[45] Kverndokk S, Melberg HO (2021). Using fees to reduce bed-blocking: a game between hospitals and long-term care providers. Eur J Health Econ..

[46] Hellesø R, Fagermoen MS. Cultural diversity between hospital and community nurses: implications for continuity of care. Int J Integr Care. 2010;10(1):e036. 10.5334/ijic.508 PMC2858515 20422021

[47] Romøren M, Pedersen R, Førde R (2017). One patient, two worlds–coordination between nursing home and hospital doctors. Tidsskr Nor Laegeforen..

[48] Bendix Andersen A, Beedholm K, Kolbæk R, Frederiksen K (2018). When clock time governs interaction: how time influences health professionals’ Intersectoral collaboration. Qual Health Res.

[49] Batalden P. Getting more health from healthcare: quality improvement must acknowledge patient coproduction—an essay by Paul Batalden. BMJ. 2018;362(8166):k3617.

[50] Kvæl LAH, Hellesø R, Bergland A, Debesay J (2022). Balancing standardisation and individualisation in transitional care pathways: a meta-ethnography of the perspectives of older patients, informal caregivers and healthcare professionals. BMC Health Serv Res.

[51] Bragstad LK, Kirkevold M, Foss C (2014). The indispensable intermediaries: a qualitative study of informal caregivers’ struggle to achieve influence at and after hospital discharge. BMC Health Serv Res.

[52] Gerring J (2004). What is a case study and what is it good for?. Am Polit Sci Rev.

